# Intangible cultural heritage: a benefit to climate-displaced and host communities

**DOI:** 10.1007/s13412-021-00697-y

**Published:** 2021-05-08

**Authors:** Gül Aktürk, Martha Lerski

**Affiliations:** 1 Department of Architecture, Delft University of Technology, Delft, 2628BL the Netherlands; 2 Leonard Lief Library, Lehman College, New York, NY USA

**Keywords:** Climate displacement, Climate justice, Intangible cultural heritage, Climate relocation, Climate adaptation, Stakeholder involvement

## Abstract

Climate change is borderless, and its impacts are not shared equally by all communities. It causes an imbalance between people by creating a more desirable living environment for some societies while erasing settlements and shelters of some others. Due to floods, sea level rise, destructive storms, drought, and slow-onset factors such as salinization of water and soil, people lose their lands, homes, and natural resources. Catastrophic events force people to move voluntarily or involuntarily. The relocation of communities is a debatable climate adaptation measure which requires utmost care with human rights, ethics, and psychological well-being of individuals upon the issues of discrimination, conflict, and security. As the number of climate-displaced populations grows, the generations-deep connection to their rituals, customs, and ancestral ties with the land, cultural practices, and intangible cultural heritage become endangered. However, intangible heritage is often overlooked in the context of climate displacement. This paper presents reflections based on observations regarding the intangible heritage of voluntarily displaced communities. It begins by examining intangible heritage under the threat of climate displacement, with place-based examples. It then reveals intangible heritage as a catalyst to building resilient communities by advocating for the cultural values of indigenous and all people in climate action planning. It concludes the discussion by presenting the implications of climate displacement in existing intangible heritage initiatives. This article seeks to contribute to the emerging policies of preserving intangible heritage in the context of climate displacement.

## Introduction

The threats of climate change—with weather changes including drought, desertification, land and forest degradation, wildfires, floods, and sea-level rise—force people in vulnerable areas to move. As a response to the threats of climate change, climate adaptation strategies focus on resist, retreat, or rebuild (Scott and Lennon 2020). In the case of retreat, climate displacement has been discussed in many different forms, including forced and voluntary, temporary, and permanent, internal and cross-borders. If planned, this strategic movement is known as managed retreat and recognized as a type of climate adaptation strategy (Hino et al. 2017). Psychological consequences of relocation in planned forms on the Pacific Island communities in the past revealed why it is the last option to consider after catastrophic disasters (Ferris and McAdam 2015). An exception to this is the choice of adaptation of low-lying islands such as Tuvalu in Oceania, whereby local stakeholders exert their agency and existing cultural and political resilience in evaluating risks and choosing not to relocate in advance of submersion (Roy 2019; Steffens 2019).

In the existing literature, use of terminologies, i.e., displacement, evacuation, planned and forced relocation, and resettlement, and their legal implications in policymaking are conflictual. Drivers associated with displacement range from environmental, e.g., drought; social, e.g., limited access to education; political, e.g., weak governance; and economic, e.g., poverty (Anzellini et al. 2020). Among the aforementioned factors, climate change is increasingly driving human displacement. The term relocation often describes the physical process of moving people permanently which can be forced or voluntary (Ferris and McAdam 2015). Displacement, on the other hand, refers to forced movement temporarily or permanently within borders or across borders (Sarah Opitz Stapleton et al. 2017). Planned relocation is a government-led action to provide protection for the displaced communities. However, the terms of displacement and relocation are often used in the moving of infrastructures, settlements, and households rather than customs, beliefs, and other cultural values. Resettlement is a more inclusive term—meaning the process of moving people with their socio-economic conditions in addition to their relocation (Ferris and McAdam 2015). Managed retreat as part of a planned relocation is often used in the context of coastal zones to mitigate against the threats of inundation, flooding, and coastal erosion. To avoid political, economic, and conflictual meanings, in this paper we identify climate-displaced peoples as such, rather than as climate migrants or refugees.

While voluntary migration may seem to be merely for the physical survival of communities, intentional policies are necessary to address successful social and cultural adaptation. Even as a deliberate act of intervention, planned relocation has often revealed challenges for communities who abandoned their homelands, leaving their sources of income, customs, habits, and memories behind. The relocation of climate-displaced people is a complex matter that heavily involves issues of human rights (Bettini et al. 2017), psychological impact of loss and grief (Torres and Casey 2017), and identity (Farbotko et al. 2016). Human costs of migratory flows associated with climate change are often interconnected. Although the anticipated results of losing the physical environment are evident, the intangible values, i.e., local traditions, identity, attachment, and sense of place along with other human dimensions, receive less attention than tangible heritage in climate displacement (Kim 2011).

Intangible cultural heritage (hereafter called ICH) is defined by UNESCO as “oral traditions, performing arts, social practices, rituals, festive events, knowledge and practices concerning nature and the universe or the knowledge and skills to produce traditional crafts”(UNESCO 2003). However, there is an underappreciation regarding many forms of ICH which are outside the label of “authorized heritage” (Smith et al. 2011) particularly in the context of climate adaptation planning efforts. Though much of the heritage is institutionalized by UNESCO through inter-governmental agreements (Batisse and Bolla 2005), there are various forms of ICH which still hold cultural significance in understanding the values, meanings, memories, and past narratives of communities (Aktürk 2020). Loss of physical landscapes results in discontinuation of cultural knowledge, traditions, customary, and folkloric practices (Aktürk and Hauser 2021). ICH is associated with landscapes, sense of place, attachment, and identity—thus should be an integral component of climate adaptation planning (Henderson and Seekamp 2018). Therefore, giving value to intangible heritage in climate displacement policymaking will assist community resilience post-retreat.

This paper briefly (1) reviews the existing literature on the intersection of climate displacement and intangible cultural heritage, (2) presents examples of the loss of ICH in the context of climate relocation, and (3) suggests the contribution of ICH in constructing long-term adaptation of displaced communities in host countries. The aim of this policy paper is to illuminate the potential role and significance of ICH in climate displacement in light of existing policies and initiatives. We briefly examine a global range of ICH—from inscribed Representative List, to Urgent designations (UNESCO), to the unlisted. We emphasize developing country populations, as these are less likely to afford costly adaptation measures. The paper reveals the significance of ICH in supporting the inclusion of displaced communities in host countries and argues that its protection, inclusion, and integration can improve climate adaptation strategies.

## Climate relocation and intangible cultural heritage

The impacts of climate mobility on the intangible values of displaced communities are rarely mentioned in the climate-relocation debates. ICH and its invaluable legacy of custodial knowledge is at risk. ICH historically interconnects with natural cultural landscapes and tangible built heritage (von Droste et al. 1995). It encompasses traditional music, dance, medicines, food, clothing, languages, oral traditions, and many other representations of cultural identity. As scales of heritage conservation broaden from objects and buildings to ecosystems, the forms of ICH expand greatly by including biocultural heritage. Standing at the intersection of culture and nature, biocultural heritage is recognized as “living organisms or habitats whose present features are due to cultural action in time and place” (UNESCO 2008). With eroding environments, the endangerment of ICH reemerges, particularly in the context of climate relocation. With it, land and landscape memories, and know-how practices of indigenous people—known as biocultural heritage—become prominent in facing climate change.

In the face of environmental change, some communities have lived with sea level rise, salinization, and flooding—have adapted to it—and integrated water into their daily lives, particularly in certain coastal and riverine cities; other communities have relocated. Stakeholders including the local council, community leaders, civil society organizations, individual residents, and property owners contribute toward building community resilience to cope with the water challenges. In this sense, those who stayed in their ancestral homes used inherited local knowledge of water management to adapt to it by terracing for agriculture, collection of rainwater in cisterns, channels, basins, and drainage systems (Jinapala and Somaratne 2002). In Dhaka, Bangladesh, a study of low-income squatter settlements impacted by flooding demonstrated that keeping stakeholders’ needs in mind can assist with internal relocation (Rashid et al. 2007). In rural coastal Bangladesh, local residents have historically built homes and homesteads to withstand regional climatic effects from tornadoes, floods, and erosion. Some modern policies are maladaptive and fail to recognize existing local adaptive knowledge. Palmyra and fishtail palms, coconut and date trees, and screw pines are among disaster-risk species once managed through traditional knowledge (Ataur Rahman and Rahman 2015), and ecological knowledge may also prove adaptive in new locales and to host communities.

Stakeholders already grappling with the fragility of agricultural, medicinal, linguistic, or spiritual traditions attempt to preserve community knowledge. For instance, the Kumeyaay Garden at the University of San Diego is a small-scale effort to preserve Native American intangible biocultural knowledge (USD University of San Diego The Kumeyaay Garden 2021). On a broader scale, land tenure traditions and irregularly enshrined in laws often embrace intangible biocultural practices. LandMark estimates that indigenous and community land maps currently cover roughly 12% of the world’s land, out of an estimated 50% or more that is held globally by indigenous peoples (LandMark: Global Platform of Indigenous and Community Lands 2020). Further research into, and understandings of, practices associated with alternate land management traditions is necessary ahead of climate migrations. Support mechanisms such as financial incentives for the documentation of cultural heritage of climate-displaced communities can help continuity of their cultural practices and traditions in the host locations (Herrmann 2019).

Climate displacement has been closely associated with land, sense of place, and identity (Adger et al. 2013; Adger et al. 2011; Quinn et al. 2015). The climate-driven loss of land—and with it, traditional economic practices including fishing, trade, and farming—inevitably causes climate-displaced communities to lose aspects of their cultural practices. Customs associated with the land, i.e., celebrations, clothing, burials, food, hunting, and farming, are underrepresented in contextual planning for climate relocation. Customary land ownership traditions, for instance, are among practices that may not be recognized by host countries. Losing their sacred places, kinship, and networks, displaced peoples also often encounter language barriers; the latter further complicates adaptation, as individuals, families, and communities use memories and narratives as vehicles to navigate their resettlement struggles.

The UN’s “Report of the Special Rapporteur in the Field of Cultural Rights” (Bennoune 2019) promotes the documenting, monitoring, and analysis of risks of holistic impacts of disruption of ICH traditions. While many communities may not be aware of Heritage Lists by UNESCO, or may not have the leisure or resources to file a rigorous application to list their ICH, examples from the formal list give a sense of the range of traditions—many of which are already vulnerable—and will be endangered under displacement situations (UNESCO 2020b). Al Azi, the Art of Performing Praise, Pride and Fortitude Poetry in the United Arab Emirates—inscribed in the List of “Intangible Cultural Heritage in Need of Urgent Safeguarding”—is endangered due to the abandonment of tribal customs and creative art practices following an internal movement from deserts to urban areas (UNESCO 2020a).

Traditional knowledge and cultural practices are under the threat of loss, appropriation, or exploitation—even in communities that have not relocated. The stresses on cultural heritage, particularly intangible values, will likely increase with relocation efforts of communities. The notion of cultural hybridization with globalization comes from mixing and synthesizing of cultural products that generate a new form of cultural element (Wang and Yeh 2005). Pressures to accept hybridization may increase as well: in adding tango to UNESCO’s “Intangible Cultural Heritage List,” the agency traced the performance art’s authentic origins to specific communities in Argentina and Uruguay, distinguishing the original dance from its iterations in places such as Japan or Finland (Bortolotto 2016). In climate relocation, hybrid identities and cultural values may emerge among both migrants and host residents in host communities.

Cultural appropriation and exploitation may arise as climate migrants attempt to adapt their traditions in alternate settings. Cultural appropriation is regularly accompanied by misrepresentations of culture, “whereas valuing a culture should involve increased sensitivity to the injustices faced by its members” (Matthes 2016). Subjected to climate relocations, certain cultural practices may be undermined or misinterpreted—or individuals, families, or communities may become alienated from traditions; this would increase tensions between the relocated and host communities.

**Fig. 1:**
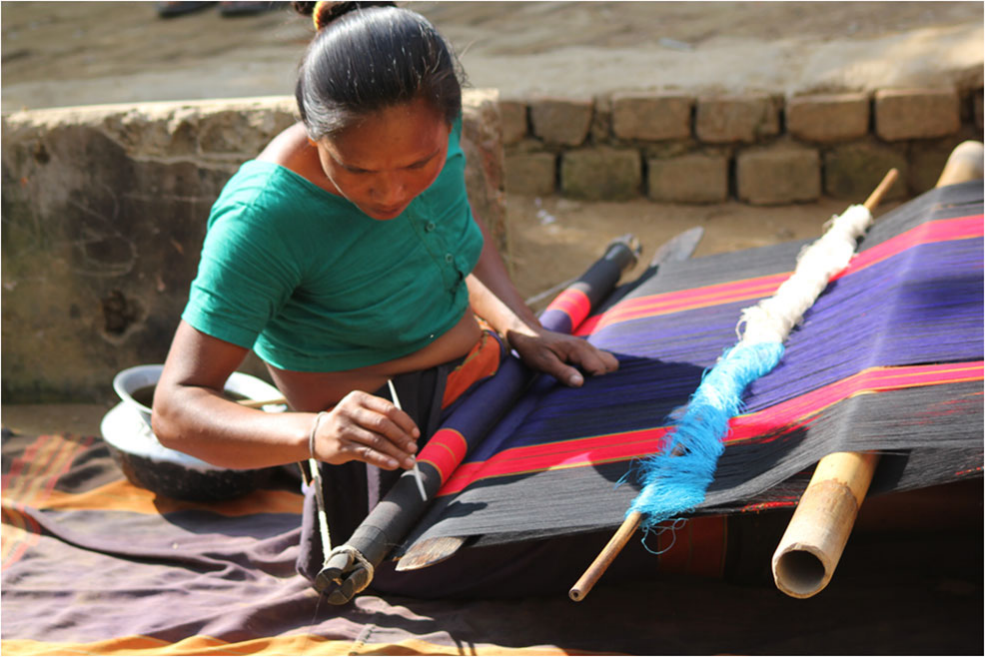
A tribal (chakma) woman making her cloth. Photo by Hasib Wahab taken on 25 December 2012, Flickr (https://flic.kr/p/dR3rnM). CC BY-NC-ND 2.0. Accessed 1 January 2021

Both physical displacement of groups and appropriation of traditions pose threats to communities’ cultures. The term “climate gentrification” is used to describe the dislocation of all but the most wealthy residents; this can combine with “gentrification,” the pushing out of middle and lower income people by higher income groups. These pressures affect the built heritage along with the associated community values (Wiggins 2018). The ben custom—loom tradition of Chakma people (Fig. 1) in Bangladesh—embodies collective intellectual property (Chakma 2020); this tradition, however, is at risk due to mass production and is reproduced without the permission of indigenous communities (Chakma 2020). Such issues have been studied in relation to globalization as well as climate change (Bortolotto 2016; Latour 2018). Migrants whose traditions and “ethos” are not welcomed by host communities possibly “remain the mindset of exile, not citizens” (Scarre et al. 2019).

Some local stakeholders exercise caution concerning external marketization (Bortolotto 2016), and communities protect against commodification, patent usurpation relating to traditional knowledge of Ayurveda, Unani, and Siddha medicinal sciences (Chakravarty and Mahajan 2010), or other intangible cultural heritage traditions (Climate and Traditional Knowledges Workgroup 2014). A challenge facing global communities is how to balance host community concerns, integration, broad appreciation for imported traditions, and respect for the integrity of said cultural heritage.

One 21st Century integration challenge arose when two million people, responding to wars, water scarcity, and food insecurity migrated to Europe from Syria and Iraq; anti-immigrant movements grew as a response to refugees. The “Ecological Threat Register” notes that while this inflow represents under half of one per cent of the European Union’s total population, that dislocation, “fueled the rise of new political parties, increased hostilities to immigrants and heightened political instability” (Institute for Economics and Peace September 2020). The effects of conflict displacement in the Syrian case include grieving, mourning, and other collective activities and events; among ICH elements which help the displaced people to recover are traditional artisanship, food, healthcare, and mourning practices (Chatelard 2017).

Huang and Hou reveal a community’s capacity to creatively maintain traditions post disaster while adapting through “relocated authenticity” (Huang and Hou 2018). Here, indigenous Taiwanese migrants pursued unexpected options, such as the crossing of class barriers via the process of modifying buildings with alternate materials.

The coronavirus pandemic shows broad potential for technology to bridge some spatial communication divides, signaling possible tools for climate adaptation. A paper on “Transnational Cultural Events Among Korean Immigrants in the New York-New Jersey Area” (Min 2017) illuminates technological opportunities towards supporting immigrant adaptation and integration in cases where a homeland remains vibrant and viable—and when the Internet, media, and technological innovations are available to enable transmission of music, drama, and dance and performances.

Successfully relocating individuals or communities involves creating avenues for the inclusion of multiple nuanced cultural elements. How do certain aspects of cultural heritage transfer, for instance, those based in agriculture? In 2010, UNESCO identified Michoacán cuisine of Mexico, which includes indigenous and Spanish traditions, a “Masterpiece of the Oral and Intangible Heritage of Humanity.” Ingredients in one region differentiate the cuisine from that of other parts of the country and are place-based; in Michoacán, these include corn, blackberries, zapote, tamarind, avocados, whitefish, and chiles (Fussell 2011; Quintana et al. 1986). What happens to climate-displaced peoples when they are separated not only from the local ingredients, but from the festivals, sports, games, music, and textures that represent intangible cultural heritage? How far are host communities prepared to support practices of traditions and to facilitate economic agency or the adaptation of migrants’ cultural skills? When migration issues are not included in governmental climate displacement policies, cultural heritage organizations, the private sector, and representatives of civil society should actively bring these matters into cultural heritage platforms.

Even planned relocations within national boundaries pose challenges to livelihoods, but cross-border movement will create additional struggles for the integration of displaced communities. Economic opportunities and labor markets continue to be roadblocks to integration in host countries (Zetter and Ruaudel 2018), and alternate citizenship or residency approaches might be considered (Milanovic 2016). Integration options should systematically consider the livelihoods of displaced people. Loss of identity extends beyond detachment from place, to include social disintegration at an individual level.

Although economic participation of newcomers is important in building community resilience and contributing to a sense of inclusion and complementing the feeling of more “homelike” settlement, cultural expressions of relocated people are equally significant (Huang and Hou 2018). Therefore, climate change policies, agreements, and initiatives should highlight the importance of just transition—which extends to the health, human, and civil rights of vulnerable communities for a safer and better future (Cahill and Allen 2020). With planning, synergies and common or complementary benefits can emerge among host and relocated groups.

Language is a proxy for culture (Lempert 2010; UNESCO 2017) and is a way of transferring knowledge—yet languages are under threat in displacement. Recognition of the central role of language in preserving and communicating culture must be a part of climate adaptation. Language-based traditions such as poetry or humor are particularly vulnerable in displacement and should receive appropriate attention and support. When it comes to accommodating people in the face of climate crisis, linguistic expressions are vulnerable to displacement but are critical to integration processes. Linguistic diversity supports the continuation of indigenous practices. For instance, Vanuatu—an island in the Pacific Ocean on the forefront of cyclones and floods—has a language diversity which comprises over 100 indigenous languages (Riehl 2019). With the destruction of infrastructures and villages, the communication network is under the threat of loss. Language assists the transmission of local and traditional knowledge through generations and thus supports marginalized groups in rebuilding their communities in host countries (Riehl 2019).

Differing scales of migration may require varied approaches. With massive population movements in climate change relocations, community-wide solutions may be more difficult. Research of two successful, small-scale, community-driven, and culturally sensitive relocations—in Alaska and the South Pacific—found that residents viewed *moving as a community* “the most important right to protect” (Bronen 2014) though one group specified remaining a “distinct, unique community,” while the other planned integration with a host community. In Taiwan, a migrant in a planned relocation site within the country noted that “where the community is where the authentic tribe is” (Huang and Hou 2018). This preference for relocation as a community raises questions about how collective movement could be planned and implemented on a large scale.

## Intangible heritage for the integration of climate-displaced communities

There have been several transnational efforts of UN, its advisory bodies, and NGOs regarding climate action and relocation, although direct and indirect benefits of intangible cultural heritage are not emphasized. UNHCR (the United Nations High Commissioner for Refugees), UN, UNFCCC (United Nations Framework Convention on Climate Change), IOM (International Organization of Migration), the Pocantico Call to Action on Climate Impacts and Cultural Heritage (Pocantico Center of the Rockefeller Brothers Fund 2015), the Sendai Framework, the Nansen Initiatives, the EACH-FOR project of the European Commission, and the Manila Initiative are some of the prominent initiatives in the international arena. Although climate-displacement has been raised as an issue since the 1951 Refugee Convention, cultural heritage has not, until this century, been explored in proportionate measure to its relevance to climate change. While cultural heritage has been flagged increasingly in reports on climate relocation, there has been inadequate emphasis on the cultural heritage of displaced communities.

**Fig. 2:**
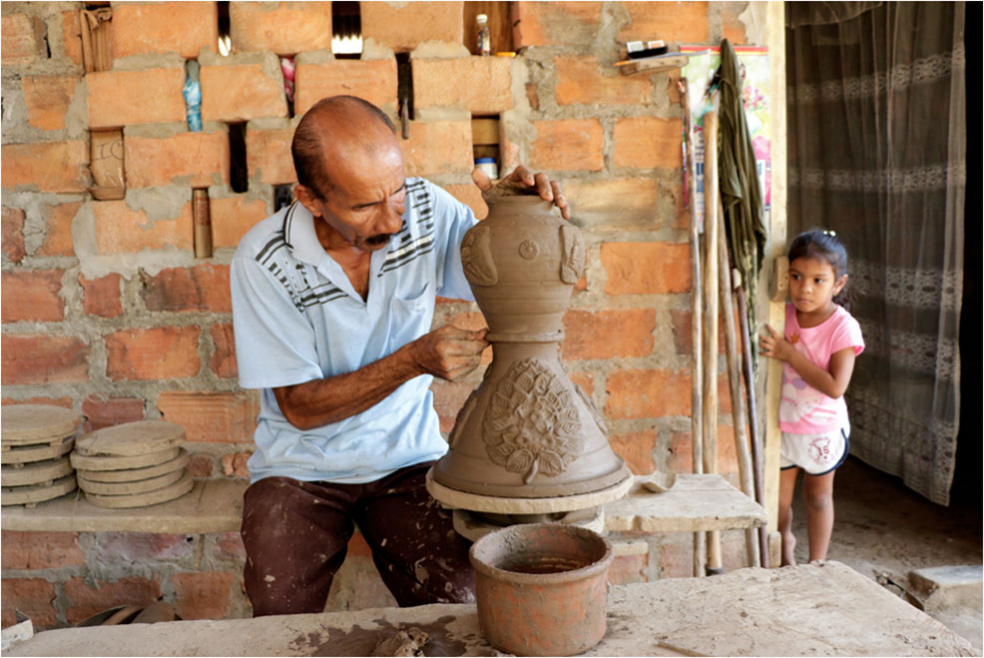
Master potter Herberto Ramírez, the last potter of Mompox, has dedicated himself to the transmission of his knowledge among the new generations, so this traditional craft does not disappear with him, © UNESCO, https://ich.unesco.org/en/BSP/safeguarding-strategy-of-traditional-crafts-for-peace-building-01480

UNESCO’s rigorously selected Register of Good Safeguarding Practices includes existing examples, and best practices, of community or government actions to preserve ICH. For example, one practice identified by UNESCO is that of the non-government organization, the Methodology for Inventorying Intangible Cultural Heritage in Biosphere Reserves; this Spanish instance was inscribed into UNESCO’s lists, and its practices can be implemented in other countries. Another safeguarding practice selected for this list is Safeguarding Strategy of Traditional Crafts for Peace Building in Colombia, which aims to train future generations to learn building crafts and skills (Fig. 2) through a workshop and by implementing policy on traditional crafts (UNESCO 2020c). For the monitoring process of the artisanship under the threat of loss, citizen science and community engagement can advocate for the use of traditional and indigenous knowledge (Bennoune 2020).

Whose heritage elements will survive depends on how resettled groups are integrated in receiving communities. Cultural values embedded into climate change policies can contribute to the preservation of the material and immaterial values of vulnerable populations (Wiggins 2018). Progress must be made in understanding the challenges and the potential benefits brought by migrants relocating in response to climate changes. During a time with increasing global tensions regarding immigration, it is important to stress to host communities that diversity is a benefit—contributing more viewpoints (Latour 2018) and problem-solving approaches in workplaces and in communities (Rock and Grant 2016).

Recognizing the intangible values and identities of displaced communities can play a significant role towards resilience and cohesion for stakeholders in relocation attempts. Displaced and host communities should find synergies from their shared values—which may evolve into “heritage” in the future. The editors of *Cultural Heritage, Ethics and Contemporary Migrations* suggest that “heritage can help displaced communities to construct meaningful futures for themselves, building on their past traditions and achievements” (Scarre et al. 2019). Echoing Min, they argue that immigrants who are receptive to the cultural heritage of their old home and their new can enjoy what both have to offer (Min 2017). Furthermore, the past cultural practices of collaborative communities can be adopted by host communities that may be more individualistic—towards creating a respectful unified heritage.

Both the end-of-year ICH and Resilience in Crisis (Intangible Cultural Heritage Courier of Asia and the Pacific 2020) and UNESCO Fifteen Session of the Intergovernmental Committee for the Safeguarding of the Intangible Cultural Heritage (UNESCO 2020d) alternated “Living Heritage,” for ICH. In the background note for its 2020 ICH NGO virtual Conference, ICH is described this way: “These days, it is called as living heritage, which emphasizes the characteristics of a source of resilience, inspiration, joy, and solidarity (Intangible Cultural Heritage Courier of Asia and the Pacific 2020).”

The immaterial cultural expressions of indigenous and minority groups are now under the threat of loss, appropriation, and exclusion in times of climate displacement. Intangible cultural practices figure prominently in collective identity and activities which bring individuals and communities together in celebrations, mourning, wedding ceremonies, musical festivals, and other traditions. At an annual UNESCO meeting to evaluate new ICH elements, the ministers from Algeria, Mauritania, Morocco, and Tunisia celebrated the formal inscription of knowledge, know-how, and practices pertaining to the production and consumption of couscous. They noted that couscous was “more than a dish”—rather a regional “marker of the culture of Maghreb in mourning and celebration”—and “an element of culture” which recognizes a “common identity,” among different nations in a region (UNESCO 2020d).

Heritage is also a possible avenue for migrants’ economic sustainability. In Tucson, Arizona, Mexican-born producers of artisanal tortillas demonstrate resilience through the practice of their culinary craft (Alvarez 2016). The First People’s Fund, and Master-Apprentice grants through the National Endowment for the Arts are examples of support that can help relocating individuals to both continue home traditions as well as maintain economic resilience. Assistance with education and job planning should accompany proactive efforts to lessen adverse cultural and psychological effects of migration (de Sherbinin et al. 2011).

In these times of hard bordering, stakeholders’ role is to work towards recognizing and acknowledging potential contributions of the relocated individuals and groups—and the latter’s role in host communities. Can traditional knowledge or practices help displaced communities to adapt to climate change in resettlement, or will climate displacement erase this knowledge? Perhaps intangible heritage’s very ephemerality, unlike archeological place-rooted remains, may strengthen its resilience through flexibility; the 2003 UNESCO Convention *emphasized communities, not places*, “thus promoting dynamic representations of culture and identity” (Bortolotto 2016).

**Fig. 3:**
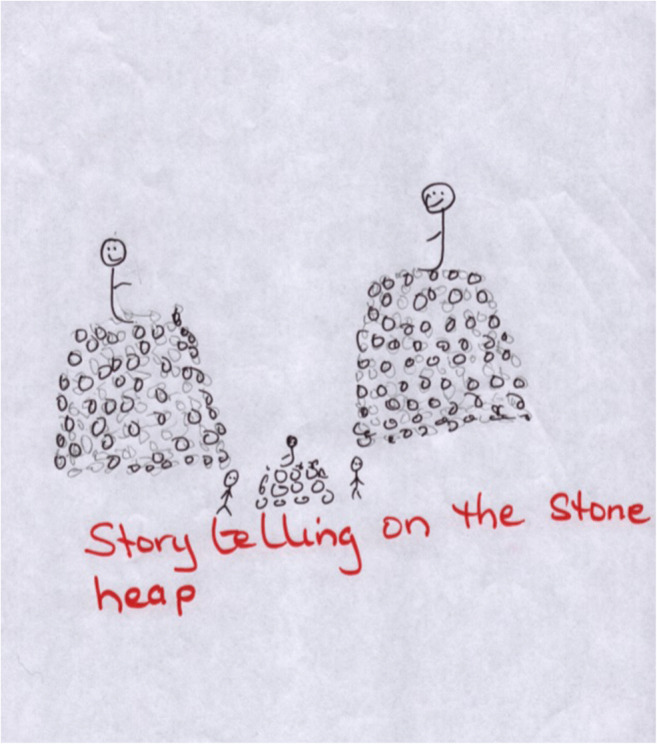
Storytelling on a stone heap. The image is courtesy of Martha Lerski

Migrants’ appreciation of landscape-based notions of intangible heritage may be transferable and assistive towards adaptive relocation. Displaced Somali women used gardening as a “conduit” for storytelling in resettlement (Coughlan and Hermes 2016); this finding resonates with cultural heritage research done in the Caribbean, where—after returning home from hurricane induced displacement—residents reflected on the fragility of their cultural traditions. In Barbuda, though the “storytelling on a stone heap” tradition (Fig. 3) is a practice that is dying out, residents wished to perpetuate their conduit through other means, in order to continue transferring knowledge intergenerationally through storytelling (Lerski 2019).

ICH should be included deliberately in adaptation plans for managed retreat, for the benefit of both relocating and host communities. The latter will adopt the customs, food, and traditions of arrival communities (Scheffler 2007). Can place-displaced traditions form a new type of intangible heritage or strengthen social inclusion of climate displaced communities? Dramatic changes to local environments and small- and large-scale movements of human populations have occurred throughout prehistoric and recent times (de Sherbinin et al. 2011; Kirch 2005), but contemporary planned relocations can benefit from both scientific climate modeling and a growing understanding about the significance of heritage traditions to communities. In addition, cultural policies and inclusion of traditional knowledge approaches can assist with best practices of protecting intangible heritage in situ or in displacement (Herrmann 2017).

Both relocated and host stakeholders should craft culturally appropriate approaches—as well as participate in mechanisms (de Sherbinin et al. 2011). Residents of the atoll Carteret Islands placed high importance on preserving their traditional fishing grounds when relocating, while the Newtok Traditional Council in Alaska emphasized that development reflect their Yup’ik cultural traditions, with local input throughout (Bronen 2014).

Local stakeholders in Guatemala participated in selecting and resettling a new city, Chuk Muk, for the Tz’utujil people. The successful relocation included cultural, ethnic, and social concerns in planning and implementation, such as designing houses to accommodate extended families. The process also considered the “indigenous cosmic world view, ethics and understanding of nature and disasters” (Cantero 2011). Preservation of “knowledge and practices concerning nature and the universe” (UNESCO 2003) should be a priority in relocation planning, requiring input from stakeholders. The Chuk Muk plan articulated a goal of integration with other cultural groups (Cantero 2011), which also contributed to host stakeholder buy in.

On an individual scale, artisans with traditional knowhow and self-taught skills face social integration- uncertainty related to the transferability of their handicrafts. The traditional crafts that are dependent on local materials may or may not be able to adapt to the local resources of the host communities. Host communities must be open to new types of cultural expression. Relocated communities have, for instance, contributed to host societies by introducing food production or cuisine or their labor power or expertise into agriculture and trade.

Some intangible heritage is less easily transferable. In Haiti, spiritual traditions are at risk because the material used for drum making, mahogany, is depleted, and even substitute woods are scarce. An elderly drum maker supports this local intangible performance tradition, and his instruments have been played at the country’s sacred sites where thousands of local and international pilgrims participate annually. The local tradition combines biodiversity and cultural practices: “the tanbou (or drums) are routinely imagined at the center of Haitian experience and provide necessary access to the spiritual forces of the universe” (Dirksen 2019).

Intangible traditions are vulnerable within national boundaries and also when displacement is temporary. Both before and after category five Hurricane Irma, interviews, surveys, and visual art created by adult and child research participants on the island of Barbuda revealed some different cultural norms than those of their politically affiliated sister island, Antigua. Land tenure traditions emerged as a key difference, relating to Barbuda’s communal ownership and fishing, hunting, and food gathering traditions. Though of the same ethnic and religious background, and sharing many common celebrations and cuisines, the two islands’ approaches to governance, food gathering, land clearing, and wildlife differ. Though returning to physical devastation and destroyed homes, Barbudans were eager to return to their place-specific cultural ways (Lerski 2019), and the brutal disrespect for Barbudan traditions and rights was described as a “cautionary tale” at the Cultural Heritage Partnership to Enable Ambitious Climate Action, at the UN Summit (United Nations 2019).

Looking to how host populations stand to benefit from the introduction of new cultural traditions, there is a possibility of individual and community affinity— “not through identity but despite difference.” Philosopher Appiah suggests that “we can only fully respond to ‘our’ art if we move beyond thinking of it as ours and start to respond to it as art” (Appiah 2009) via a common heritage of creativity that transcends place and the tangible. Far from placing individuals or communities in static silos of place or tradition, intangible cultural heritage, as described by UNESCO, bolsters social cohesion and responsibility towards both local and broader communities—and plays a role in “bringing human beings closer together and ensuring exchange and understanding among them” (UNESCO 2003).

ICH sustains diverse identities and supports the displaced in a sense of belonging. Intangible values can assist emotional integration of the displaced, in addition to potentially introducing ecological traditional knowledge practices. Among intangible cultural elements are alternate world views, including reciprocity—a far distance from a colonizing approach to building community or the environment. With a stated goal of consensus, representatives of Native American Ojibwe and Menominee tribes—along with academic, intertribal, and government entities—designed a framework in which cultural inclusion is prioritized when developing a roadmap for climate adaptation; culture here explicitly includes relationships with landscapes and non-human beings (Tribal Adaptation Menu Team 2019).

Supporting social cohesion, ICH can enhance the resilience of communities in times of climate crisis. Domination of host communities over climate-displaced communities may result in placing host culture over the culture of displaced communities. Oppressed communities require access to and protection of their intangible values. Thus, though ICH of displaced communities is more vulnerable to climate change, it is a powerful tool for adaptation as well as reciprocal benefit. Intangible heritage policies should be included in climate-displacement action plans—both for the protection and transmission of knowledge, culture, and social cohesion of migrants and for the benefit of host communities—towards successful integration and avenues for mutual understandings.

Complementing scientific data on environmental change, traditional knowledge, cultural heritage, art, and creativity of indigenous communities can inform decision-makers towards developing and shaping better adaptation policies and practices (United Nations 2020). Inclusion of cultural initiatives in climate action efforts can advocate humanitarian, ethical, and social aspects of preservation of intangible cultural heritage. Therefore, building the bridge between cultural heritage actors and environmental agencies can help host and displaced communities to benefit from this cultural exchange and diversity.

## Conclusion

Climate change impacts both developing and developed countries which suffer from drought, sea-level rise, coastal erosion, and wildfires—leading to climate-displacement. Confronted with climate-induced loss of lands, water, and livelihoods, communities are displaced voluntarily or forcefully. From small developing island states to African climate migrants, communities suffer not only from economic and environmental vulnerabilities but also endangerment of their cultural values. ICH has already been under the threat of globalization with the abandonment of localized knowhow skills and inherited knowledge. However, the climate crisis multiplies the risks on ICH elements, as it threatens individuals’ and communities’ close relationships with land, people, the environment, and tradition. Different resilience capacities among nations impact the ability of host communities to cope with the integration refugees (Institute for Economics and Peace September 2020), but recognition of ICH towards successful adaptation and healing offers benefits to both host communities and migrants.

The dominance of the intangible values of host communities over the identity and culture of vulnerable communities may cause corruption of social integrity if vulnerabilities of the climate displaced are not considered. Cultural *appreciation* is an inclusive approach to the cultural values of displaced people in social inclusion and integration. In addition, opportunities for both displaced migrants and host communities may be wasted if multiple stakeholders do not deliberately and immediately plan with attention to the benefits and the vulnerabilities associated with intangible heritage in climate-associated displacement.

Some relocations result in new forms of heritage—for instance, changing to land from ocean-based food traditions or achieving successful relocation by otherwise adapting to new local conditions, as local resources change with relocation, labor, and intangible cultural heritage, i.e., craftsmanship, language, and food adapt into new settlements. If host communities welcome interaction with the values of displaced communities, a changing heterogeneous society can be achieved. How can policies ensure the preservation of intangible values of socially vulnerable communities who are displaced from their homelands? Just as the formal UNESCO processes for nations as well as NGOs have specific guidelines for applicants and participants, nations and regional and international organizations should have specific recommendations and markers for evaluation, proactive actions, and mitigations.

Successful intangible heritage examples can serve as templates for adaptation. Whether addressing internal or external displacement, ICH offers possibilities to support a just transition towards community resilience and positive environmental outcomes. Reciprocity of knowledge systems and funding and policy mechanisms can support displaced peoples and host communities. Initiatives for locals to revive their biocultural knowledge are also a way to support their economic livelihood. Displaced people can contribute time-tested environmental approaches (Herrmann 2017). Traditional knowledge systems and voices of marginalized groups may also offer alternative economic and policy perspectives for global challenges, such as modified energy or consumption approaches (Munshi and Kurian 2020).

The role of cultural heritage before, during, and after climate-displacement has been underlined in the International Council on Monuments and Sites(ICOMOS) report of The Future of Our Past (ICOMOS Climate Change and Heritage Working Group 2019). Resettling and reinventing in a socially, politically, and economically different environment, communities seek recognition of their intangible heritage, i.e., cultural attitudes, identity, and language. The report emphasizes the use of heritage in supporting social integrity and ensuring justice and equity among the host and displaced communities (ICOMOS Climate Change and Heritage Working Group 2019).

The current global tensions regarding migrants put climate-displaced people in jeopardy. Relocation within borders, and across districts and regions, already causes tension between host and displaced communities. In the case of cross-border movement, there is a need for increased political discourse concerning climate displacement which considers the integration of displaced populations. Challenges such as recent anti-immigration sentiments and policies by host countries (Institute for Economics and Peace September 2020) such as the USA under the Trump administration, Britain and Brexit, and anti-immigration parties in Austria, Sweden, and Hungary present additional challenges to adaptation. Environmental justice cannot be promoted without addressing the existing racial and ethnic injustices on migrants.

ICH offers unique elements to host and displaced communities in the context of climate change. Heritage that is intangible is not nation-state bound, not bound by built traditions viewed as dependent on place or a singular past. This framework does not define citizens via “perceptions of ethnicity and belonging based on a shared heritage and origin linked to blood and soil” but “is an understanding of a shared present and a shared future where sense of belonging is linked to mutual values (Högberg 2016).

This paper, like the work of Huang and Hou (Huang and Hou 2018) on “Relocated Authenticity” in Taiwan, explores how heritage in the context of relocation presents opportunities as well as threats. The 2003 ICH Convention (UNESCO 2003) encouraged vital models of identity and culture (Bortolotto 2016), and current ICH gatherings—particularly during the period of disruptions and imposed isolation of the SARS CoV-2 pandemic—point to the dynamic, “living” opportunities in intangible cultural heritage towards adaptation and community building (UNESCO 2020d; Intangible Cultural Heritage Courier of Asia and the Pacific 2020).

Like the global society at large, climate-displaced and host communities can both benefit from intangible cultural heritage. One example of non-material culture as adaptive mechanism was the transcendence of traditional national tensions in a creative solution arrived at by a Greek immigrant in the USA and a Turkish immigrant in Germany. Their cultural bridge *through the shared experience of being immigrants and scientists* alerts us to enormous possibilities for the consideration and inclusion of intangible elements in planning for climate adaptation. In this instance, a successful vaccine was created towards addressing the global COVID-19 pandemic (Gelles 2020). Supporting cultural integration of displaced peoples should be consistently included in environmental justice efforts—with funding and participation of governmental and other entities and local stakeholders.

## Data Availability

Not applicable
